# An Inquiry into the Action of Mercury on the Living Body

**Published:** 1847-04-01

**Authors:** 


An Inquiry into the Action of Mercury on the Living Body. By *Joseph Swan*. Third Edition, stitched, pp. 34. London, 1847.

Mr. Swan is a disbeliever in, though he does not absolutely deny, the absorption of mercury and its compounds. But, whether mercurials become absorbed and enter the circulation, or produce their effect by contact with living parts, they affect, in the opinion of Mr. Swan, the nervous system. The parts of the nervous system acted on are the ganglia and branches of the sympathetic nerve. By the action of mercury on them, these parts become irritated, stimulated, or inflamed.

Mr. Swan dissected the sympathetic nerve of a person "who seemed to have been under the influence of mercury," and found that the ganglia and branches of the sympathetic and the par vagum were enlarged. He made twelve experiments to determine the effects of mercurials on animals, and found, after death, that the ganglia and sympathetic nerves were redder and more vascular than usual.

From these experiments Mr. Swan infers that mercury, when swallowed, first produces an irritation of the branches of the sympathetic nerve, spread on the villous coat of the intestines; and, by communication, may secondarily affect the nerves of the limbs, but not to the same extent as the sympathetic.

Mercury, he says, is a decided stimulant of the nervous system, and, through this, of the sanguiferous system. When it has been swallowed, it produces its peculiar irritation on the stomach and intestines, and afterwards probably passes off somewhat altered in its chemical nature; for, after the use of calomel and crude mercury, matter resembling the grey oxide has been several times found in the stools and in the intestines.

"A small quantity of mercury gently excites the salivary and absorbent organs, and promotes the functions of the viscera without any disturbance of the constitution. But, when it is continued so as to affect the body generally, it produces an increased action of the heart and arteries, a furred tongue; and, according to the vigour or feebleness of the patient, irritative fever, an increase or depression of the spirits, and generally, along with one or more of these symptoms, irritation in the mouth, and an increase of the saliva, urine, perspiration, bile, and other secretions, through the excitement it produces on the respective organs. When a sufficient quantity has been employed, it produces a general excitement of the sympathetic, and through this of the viscera."

Mr. Swan states that, independently of its specific influence, the inflammation it excites quickly tends to ulceration.

"As mercury acts so decidedly on the sympathetic nerve, it can be readily understood how the intestines and the rest of the abdominal viscera are influenced, and the heart and arteries, and how it excites the action of the salivary glands and other parts about the mouth by means of the branches of the superior cervical ganglion, which accompany all the branches of the external carotid artery in nearly the same manner as those of the semilunar ganglion do the arteries distributed on the abdominal viscera."

From the preceding extracts and statements our readers will form some idea of Mr. Swan's hypothesis of the action of mercury.

